# Psilocybin as an antidepressant strategy - a review of safety aspects

**DOI:** 10.1192/j.eurpsy.2023.679

**Published:** 2023-07-19

**Authors:** R. Zeiss

**Affiliations:** Department of Psychiatry, University of Ulm, Ulm, Germany

## Abstract

**Introduction:**

Psilocybin is considered a classical psychedelic and is increasingly attracting scientific and media attention as an alternative approach to the treatment of various mental disorders.

Apart from its efficacy, an important question is the tolerability and safety of psilocybin in general and in a controlled environment. Accurate knowledge of drug safety aspects might be essential for applicability in clinical practice and for drug adherence.

**Objectives:**

This paper aims to provide an overview of drug safety aspects of psilocybin.

**Methods:**

A narrative review was conducted. The literature search was conducted using the bibliographic database MEDLINE.

**Results:**

The literature search of papers published in recent years showed no serious side effects under psilocybin in controlled study conditions. Common reported ADRs were headache, gastrointestinal complaints such as nausea, diarrhoea and vomiting, tachycardia and arterial hypertension. The lethal dose of psilocybin is many times higher than the therapeutic dose and overdose deaths have not been identified.

An often mentioned problem is the occurrence of hallucinogenic persisting perception disorder (HPPD) which, however, did not occur in the studies examined and is most likely to be a problem in the context of recreational use. The results on the safety of psilocybin must be regarded as preliminary; in the studies conducted, risk populations were predominantly excluded, which is, however, relevant for everyday clinical practice. The risk of delusional experiences and so-called “bad trips” is also a relevant safety risk, as it can be associated with risky behaviours. However, these would also be observed more in the area of recreational use.

**Conclusions:**

The use of psilocybin in rigorously controlled study designs appears to be predominantly safe and without serious side effects. At the same time, it should be noted that the results must be considered preliminary and many questions remain open. Many of the risks are more likely to occur in uncontrolled recreational use of psilocybin. At the same time, we see a certain risk in the use of a substance associated with high expectations and a certain “fame” that, without appropriate regulations, the boundaries between sensible therapeutic use and abusive use could become blurred and permeable.

**Disclosure of Interest:**

None Declared

